# Possible mechanisms underlying intermittent synchronous activity in the networks of excitatory and inhibitory bursting neurons

**DOI:** 10.1186/1471-2202-12-S1-P276

**Published:** 2011-07-18

**Authors:** Choongseok Park, Leonid L Rubchinsky

**Affiliations:** 1 Department of Mathematical Sciences and Center for Mathematical Biosciences, Indiana University Purdue University Indianapolis, Indianapolis, IN 46202, USA; 2 Stark Neurosciences Research Institute, Indiana University School of Medicine, Indianapolis, IN 46202, USA

## 

Basal ganglia circuits in Parkinson’s disease constitute a prominent example of neural system of coupled inhibitory and excitatory neurons, which exhibit synchronous activity [1]. The phase-locking of neural activity in this system, as analyzed in experimental data intraoperatively recorded in Parkinsonian patients, exhibits intermittent temporal dynamics [2]. The mechanisms of this intermittent synchronous dynamics make the subject of this study.

**Figure 1:**
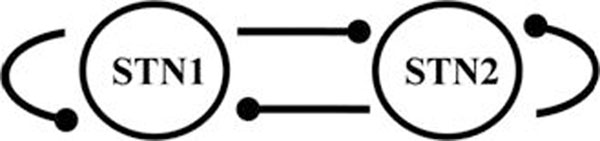
The network architecture. Each connection is inhibitory synapse.

The conductance-based network models of subthalamo-pallidal circuitry of basal ganglia have being shown to reproduce the intermittent temporal patterns of phase-locking observed in experiments with high fidelity [3]. We start with this network model of synaptically coupled excitatory subthalamic and inhibitory pallidal bursting neurons. We further reduce it to the model of two mutually coupled inhibitory neurons with self-inhibition. This network is presented at Figure 1. While real subthalamic neurons are excitatory, the resulting two-neurons network is adequate simplification of the original circuitry under assumption of high fidelity of responses of inhibitory pallidal cells. While this reduction may be associated with the loss of some other biologically realistic features, it produces a relatively generic circuit, therefore the studied mechanism may be quite general too.

We used geometric dynamical systems and singular perturbation methods to reduce the full model to a simpler set of equations. Mathematical analysis was completed using three slow variables with two different time scales. Intermittently synchronous oscillations are generated by overlapped spiking which crucially depend on the geometry of slow phase plane and the interplay between slow variables as well as the strength of synapses. Two slow variables are responsible for the generation of out-of-phase stable solution and the other slower variable for irregular and intermittent activity pattern. The results of analysis can be traced to particular values of biophysical parameters (synaptic strength and parameters of calcium dynamics), which are known to be impacted in Parkinson’s disease.
